# Editorial: Checkpoint immunotherapy: reshaping the landscape of gastrointestinal cancer treatment

**DOI:** 10.3389/fimmu.2025.1723645

**Published:** 2025-12-08

**Authors:** Stavros P. Papadakos, Elias Kouroumalis, Georgios Germanidis, Stamatios Theocharis

**Affiliations:** 1 First Department of Pathology, Medical School, National and Kapodistrian University of Athens, Athens, Greece; 2 First Academic Department of Gastroenterology, Medical School of National and Kapodistrian University of Athens, General Hospital of Athens “Laiko”, Athens, Greece; 3 Department of Gastroenterology, Panepistimiako Geniko Nosokomeio Irakleiou (PAGNI) University Hospital, University of Crete Medical School, Heraklion, Greece; 4 Division of Gastroenterology and Hepatology, First Department of Internal Medicine, American Hellenic Educational Progressive Association (AHEPA) University Hospital, Aristotle University of Thessaloniki, Thessaloniki, Greece; 5 Basic and Translational Research Unit, Special Unit for Biomedical Research and Education, School of Medicine, Faculty of Health Sciences, Aristotle University of Thessaloniki, Thessaloniki, Greece

**Keywords:** immune checkpoint inhibitors, gastrointestinal cancers, tumor regression, organ preservation, biomarkers

Immune checkpoint inhibitors (ICIs) targeting PD-1, PD-L1, and CTLA-4 have become standard treatments for advanced gastric, biliary tract, and colorectal cancers, significantly extending survival, achieving tumor regression, and enhancing organ preservation, particularly in dMMR/MSI-H subtypes (1–4). These therapies enable conversion of unresectable to resectable disease, as seen in gastric and colorectal cancers, and improve quality of life by reducing recurrence and supporting organ-sparing approaches. This editorial synthesizes contributions from recent studies in *Frontiers in Immunology*, highlighting the clinical utility of ICIs across key GI cancers, including gastric/gastroesophageal junction (GC/GEJ) cancer, hepatocellular carcinoma (HCC), biliary tract cancer (BTC), colorectal/rectal cancer (CRC/RC), and esophageal squamous cell carcinoma (ESCC). This editorial also situates these advancements within the broader immunotherapy landscape.

## Clinical utility of immunotherapy in GI cancers

ICIs have demonstrated robust clinical benefits across GI cancers, transforming treatment strategies and outcomes.

### Gastric/gastroesophageal junction cancer

ICIs significantly enhance outcomes in advanced GC/GEJ. Xu et al. demonstrated that first-line ICI plus chemotherapy extends progression-free survival (PFS) to 357 days versus 270 days for chemotherapy alone, with a disease control rate (DCR) of 38% versus 14.5% and manageable adverse events (AEs). Neoadjuvant immunochemotherapy achieves remarkable responses: Sun et al. reported the first pathological complete response (pCR) in synchronous multiple gastric cancer (SMGC) using tislelizumab plus SOX, enabling R0 resection. Similarly, Li et al. documented tumor regression in hepatoid adenocarcinoma of the stomach (HAS), reducing serum AFP levels from 52,951.56 ng/mL to 241.04 ng/mL with sintilimab plus SOX, facilitating curative surgery. Li et al. demonstrated that hyperbaric oxygen therapy (HBOT) with CAPOX and sintilimab yields a clinical complete response (cCR) in advanced HAS with peritoneal metastasis, highlighting a novel approach to overcoming therapeutic resistance in advanced gastric cancer. Yang et al. identified blood-based biomarkers (e.g., CA125, CA199, and PLR) that predict ICI response, improving patient selection. These findings underscore the role of ICIs in prolonging survival, addressing mechanisms of therapeutic resistance in GC/GEJ.

### Hepatocellular carcinoma

ICIs improve HCC outcomes when combined with targeted therapies. Liang et al. reported that postoperative hepatic arterial infusion chemotherapy (HAIC) with lenvatinib and PD-1 inhibitors significantly enhances disease-free survival (DFS) in solitary, large HCC, with no increase in hepatic toxicity, leveraging systemic immune activation to reduce recurrence. Hochnadel et al. identified novel T-cell inhibitory targets (Ngp, Hba-a1, and S100a8) via RNAi screening, with S100A8/S100A9 upregulated in human HCC, offering new immunotherapeutic possibilities. These studies highlight ICIs’ ability to extend survival and introduce novel targets in HCC.

### Biliary tract cancer

ICIs are a cornerstone of advanced BTC treatment. Zheng et al. reported a median overall survival (OS) of 15.7 months and a PFS of 8.4 months with first-line ICIs, with an 8.6% incidence of grade 3–4 immune-related AEs, indicating manageable toxicity. Durvalumab shows numerically superior OS compared to sintilimab, suggesting subtype-specific efficacy. These results affirm the robust survival benefits of ICIs in BTC, particularly in first-line settings.

### Colorectal/rectal cancer

Immunotherapy excels in dMMR/MSI-H CRC, with Deng et al. reporting a 75% pCR rate with neoadjuvant ICI therapy, particularly with dual PD-1/CTLA-4 blockade (nivolumab plus ipilimumab), which enables organ preservation. Zhang et al. extended these benefits to pMMR/MSS rectal cancer, achieving a 37% pCR rate and a 77% anal preservation rate with neoadjuvant immunotherapy, thus expanding its utility to a resistant subtype. These findings emphasize the role of ICIs in achieving tumor regression and supporting organ-sparing strategies in CRC/RC.

### Esophageal squamous cell carcinoma

Feng et al. demonstrated that adjuvant immunotherapy after neoadjuvant immunochemotherapy and esophagectomy improves DFS (38.5% vs. 23.9%) and OS (61.5% vs. 37.0%) in ypT+N+ ESCC patients, reducing the risk of recurrence. This sequential approach highlights the value of ICI in improving long-term outcomes in ESCC.

## Key research themes

### Combination therapies

Combining ICIs with other modalities enhances efficacy. Zhang et al. reported a 33.3% tumor regression grade (TRG) 0/1 rate with trastuzumab plus chemoimmunotherapy in HER2-positive gastric cancer, supporting its neoadjuvant role. Wei et al. proposed a phase II trial of nab-paclitaxel plus cadonilimab for second-line GC post-immunochemotherapy failure, exploring immune rechallenge to restore anti-tumor responses. This trial investigated potential biomarkers, such as ctDNA and TMB, to identify responsive patients. Li et al. introduced HBOT as an immunosensitizing strategy, achieving cCR in HAS by alleviating tumor hypoxia, which suppresses immune responses in the tumor microenvironment (TME). These studies demonstrate how combinations address resistance and improve outcomes.

### Biomarker discovery

Biomarker identification is pivotal for precision medicine. Wen et al. developed a four-gene liquid-liquid phase separation (LLPS) signature (DACT1, EZH2, PAK2, and PSPC1) for gastric cancer prognosis, with PSPC1 knockdown inhibiting tumor proliferation, suggesting it could be a therapeutic target. Hochnadel et al. identified Ngp, Hba-a1, and S100a8 as T-cell inhibitors in HCC. These inhibitors were validated in human samples and open new immunotherapeutic avenues. Yang et al. used peripheral blood markers (e.g., pre-IBIL, post-CA125, and CA199) to predict ICI outcomes in GC/GEJ cancer, enhancing patient stratification. These advances enable tailored treatment by identifying responsive patients.

### Cost-effectiveness

Economic analyses address ICI accessibility. Lang et al. found that cadonilimab plus chemotherapy is cost-effective for GC patients with PD-L1 CPS ≥5 in China (ICER: $37,499.27/QALY), but not without price reductions. Zhou et al. reported similar findings for HER2-negative GC/GEJ cancer, with ICERs exceeding willingness-to-pay thresholds, highlighting the need for pricing reforms to ensure equitable access, particularly in resource-limited settings.

### Systematic reviews

Zhang et al. conducted a meta-analysis of neoadjuvant immunotherapy in pMMR/MSS rectal cancer, reporting a 37% pCR rate and a 77% anal preservation rate, with short-course radiotherapy and PD-1 inhibitors outperforming alternative treatment options. This study supports the broader application of immunotherapy in challenging subtypes, complementing clinical findings with robust evidence.

## Broader context and future directions

These studies highlight the transformative impact of ICIs on GI cancer management, achieving prolonged survival, tumor regression, and organ preservation across diverse malignancies. Combination therapies, such as ICIs with chemotherapy, targeted agents, or HBOT, address resistance mechanisms, particularly in immunologically “cold” tumors, such as HAS, where hypoxia and immunosuppressive TMEs limit efficacy (Li et al., Li et al.). Neoadjuvant and adjuvant strategies, as seen in ESCC and CRC, reduce recurrence rates and enable organ-sparing surgeries, improving quality of life. Biomarker research, including LLPS signatures and novel T-cell targets, advances precision medicine, while real-world studies validate clinical trial findings in diverse populations.

However, challenges persist, including tumor heterogeneity, ICI resistance, and high costs. Heterogeneity, as seen in SMGC and HAS, complicates treatment responses due to interlesional variability (Sun et al.). Resistance, driven by immunosuppressive TMEs and low tumor mutational burden, limits efficacy in pMMR/MSS cancers, necessitating strategies such as HBOT or immune rechallenge (Wei et al.). High costs, as noted by Lang et al. and Zhou et al., restrict access, particularly in low-resource settings, requiring pricing reforms and global health policy initiatives.

Future research should focus on overcoming resistance through TME modulation. This can be achieved through HBOT, vascular normalization, or novel immune agonists to enhance ICI penetration and immune activation. Validating biomarkers such as TMB, MSI, ctDNA, and peripheral blood markers will improve patient selection, as emphasized by Yang et al. and Wen et al.,. Prospective trials, such as those proposed by Wei et al. and Li et al., are critical to confirming the efficacy of immune rechallenge and adjuncts such as HBOT Multi-omics approaches integrating genomics, transcriptomics, and proteomics will refine personalized treatment, while real-world evidence will validate these approaches in clinical practice.

## Conclusion

This Research Topic underscores the profound impact of checkpoint immunotherapy on GI cancer management, demonstrating significant clinical benefits, innovative combination strategies, and biomarkers for personalized care. By addressing efficacy, safety, and accessibility, these findings pave the way for more effective and equitable treatments, inspiring continued innovation to improve outcomes for GI cancer patients worldwide.
